# Functional Characterization of Bacterial Communities Responsible for Fermentation of *Doenjang*: A Traditional Korean Fermented Soybean Paste

**DOI:** 10.3389/fmicb.2016.00827

**Published:** 2016-05-31

**Authors:** Woo Yong Jung, Ji Young Jung, Hyo Jung Lee, Che Ok Jeon

**Affiliations:** Department of Life Science, Chung-Ang UniversitySeoul, South Korea

**Keywords:** *doenjang*, soybean paste, bacterial community, metabolites, *Bacillus*, *Tetragenococcus*, biogenic amine, GABA

## Abstract

*Doenjang* samples were prepared in triplicate and their microbial abundance, bacterial communities, and metabolites throughout fermentation were analyzed to investigate the functional properties of microorganisms in *doenjang*. Viable bacterial cells were approximately three orders of magnitude higher than fungal cells, suggesting that bacteria are more responsible for *doenjang* fermentation. Pyrosequencing and proton nuclear magnetic resonance spectroscopy were applied for the analysis of bacterial communities and metabolites, respectively. Bacterial community analysis based on 16S rRNA gene sequences revealed that *doenjang* samples included *Bacillus, Enterococcus, Lactobacillus, Clostridium, Staphylococcus, Corynebacterium, Oceanobacillus*, and *Tetragenococcus*. These genera were found either in *doenjang*-*meju* or solar salts, but not in both, suggesting two separate sources of bacteria. *Bacillus* and *Enterococcus* were dominant genera during the fermentation, but their abundances were not associated with metabolite changes, suggesting that they may not be major players in *doenjang* fermentation. *Tetragenococcus* was dominant in 108 day-*doenjang* samples, when lactate, acetate, putrescine, and tyramine increased quickly as glucose and fructose decreased, indicating that *Tetragenococcus* might be primarily responsible for organic acid and biogenic amine production. *Lactobacillus* was identified as a dominant group from the 179-day samples, associated with the increase of γ-aminobutyric acid (GABA) and the decrease of galactose, indicating a potential role for this genus as a major GABA producer during fermentation. The results of this study clarified the functional properties of major bacterial communities in the *doenjang* fermentation process, contributing to the production of safe and high-quality *doenjang*.

## Introduction

*Doenjang* is a Korean traditional soybean paste popularly consumed as a condiment for vegetables, fish, and meats or used as a seasoning ingredient in authentic Korean cuisine. The paste has received considerable attention because of numerous reported beneficial human health effects, including antioxidant, fibrinolytic, antimutagenic, and anticancer properties (Kim, 2004; Yun, 2005; Jung et al., 2006; Park et al., 2008; Namgung et al., 2009; Kwon et al., 2010; Tamang et al., 2016a).

Culture-based approaches have been widely applied to bacterial community analysis of *doenjang* (Yoo et al., 1999; Jeong et al., 2014), but they have produced limited information because culturing is time-consuming and laborious, and because *doenjang* contains unculturable microbes. Recently, culture-independent methods, such as denaturing gradient gel electrophoresis (DGGE) and pyrosequencing, have been widely used to investigate bacterial communities in *doenjang* (Cho and Seo, 2007; Kim et al., 2009; Nam et al., 2012). However, previous studies using culture-independent methods have limited their analyses to snapshots of bacterial communities by focusing on short-time frames within the *doenjang* fermentation process. To the best of our knowledge, thus far, no study has been conducted to investigate microbial community fluctuation over the full *doenjang* fermentation period. In Korea, traditional *doenjang* is typically made by further fermentation of the solid parts from a fermented mixture of *doenjang*-*meju* (fermented soybean bricks) and brine. The additional fermenting procedure also suggests that the microbial community and indigenous enzymes in *doenjang*-*meju* are likely important in determining the microbial community and metabolite change during *doenjang* fermentation. However, no research exists on how *doenjang* microbial communities alter when *doenjang*-*meju* with known microbial community composition is used.

Traditional *doenjang* is produced by spontaneous fermentation without the use of starter cultures, leading to the growth of diverse microorganisms. In turn, quality variation of *doenjang* products tends to result, as well as the occasional production of undesirable metabolites, such as biogenic amines (BAs) or toxins (Cho and Seo, 2007; Shukla et al., 2010; Park et al., 2014). Most previous studies have focused on the analysis of either microbial communities or metabolites in *doenjang* (Cho and Seo, 2007; Kim et al., 2009; Rhyu and Kim, 2011; Nam et al., 2012), which makes it difficult to investigate microbial functional properties during *doenjang* fermentation. Instead, examining microbial successions and metabolite changes simultaneously is crucial for a better understanding of microbial community function in *doenjang*. However, such studies have not yet been performed.

Pyrosequencing based on 16S rRNA gene sequences has been broadly applied to analyze microbial communities in fermented foods, because it yields more detailed data compared with conventional microbiological methods, such as DGGE and culture-based approaches (Nam et al., 2012; Park et al., 2012). Additionally, proton nuclear magnetic resonance (^1^H NMR) spectroscopy is one of the easiest, yet most comprehensive, and powerful tools to analyze diverse metabolites simultaneously in fermented foods (Jeong et al., 2013; Jung et al., 2013; Lee et al., 2015). In this study, we used pyrosequencing and ^1^H NMR techniques to investigate microbial succession and metabolite changes, respectively, across the full length of *doenjang* fermentation. The resultant data will increase our knowledge regarding the functional properties of major microbial communities involved in *doenjang* fermentation.

## Materials and Methods

### Doenjang Preparation, Sampling, and Analysis

*Doenjang* was prepared in triplicate following a traditional manufacturing method. On January 25, 2013, 90 fermented *doenjang*-*meju* bricks from a previous study (Jung et al., 2014) were placed into a large porcelain pot (called jang-dok) filled with 180 L of approximately 20% (w/v) solar salt (salts made by exposing seawater to the sun; Shinan, Korea) solution (Jung et al., 2015). The mixture of *doenjang*-*meju* bricks and solar salt solution was stored for 42 days without temperature control in a temporary structure to avoid inclement weather, and then separated into liquid and solid portions. The solid parts (*doenjang*) were mashed well and equally dispensed into three small porcelain pots, marking the start (0 day) of *doenjang* fermentation. These pots containing *doenjang* were stored in the temporary structure without temperature control for 332 days. *Doenjang* samples were intermittently collected for analysis of viable cell numbers, pH, bacterial communities, and metabolites.

Total viable cells of bacteria and fungi were estimated using a standard counting method as described previously (Jung et al., 2014). *Doenjang* samples (2 g) were resuspended and serially diluted in PBS buffer (137 mM NaCl, 2.7 mM KCl, 10 mM Na_2_HPO_4_, 2 mM KH_2_PO_4_, and pH 7.2). The diluted supernatants were spread on agar media and incubated at 30°C for 3 days. Respectively, trypticase soy agar (TSA; BD, USA) and potato dextrose agar (PDA; BD, USA), each containing 3% (w/v) NaCl, were used for bacterial and fungal cell counts. Bacterial and fungal cell numbers were counted as colony forming units (CFU) per g-fresh weight of *doenjang*.

For pH measurements, 10 mL of distilled water was added to 2 g of *doenjang* samples and vortexed, after which pH values were obtained using a pH meter (Thermo Scientific, USA). For NaCl, concentrations were measured using the Mohr method (AOAC, 2000) and expressed as a percentage (w/w) in the *doenjang* water phase.

### Barcoded Pyrosequencing for Bacterial Community Analysis

To analyze changes in the bacterial community during *doenjang* fermentation, 2 g each of *doenjang* samples were collected from the three porcelain pots and combined. Total genomic DNA was extracted from the combined *doenjang* samples using the FastDNA Spin kit (MP Biomedical, USA), following manufacturer protocol. The V1–V3 regions of bacterial 16S rRNA genes from total genomic DNA were amplified for barcoded pyrosequencing using Bac9F (5′-adaptor B-AC-GAG TTT GAT CMT GGC TCA G-3′) and Bac541R (5′-adaptor A-X-AC-WTT ACC GCG GCT GCT GG-3′) primers, as described previously (Lee et al., 2012). The “X” denotes 7–10 barcoded sequences for sorting mixed sequencing reads (Supplementary Table S1). The PCR products were purified using a PCR purification kit (Bioneer, Korea), and their concentrations were measured using an ELISA reader equipped with a Take3 multivolume plate (SynergyMx; BioTek). A pooled composite was prepared by mixing equal amounts of the purified PCR products and then sequenced using the 454 GS-FLX Titanium system (Roche, Germany) at Macrogen (Korea).

### Processing and Analysis of Pyrosequencing Reads

Pyrosequencing reads were processed and analyzed using RDPipeline tools^1^ (Cole et al., 2014). The reads were sorted into individual *doenjang* samples based on their unique barcodes, and then the barcodes were eliminated. Low-quality reads were excluded; these included sequences with more than two ambiguous base calls (“N”), shorter than 300 bp, or average quality scores below 25 (error rate, 0.005). Potential chimeric sequencing reads were also excluded using USEARCH 6.0 available in the RDPipeline (Edgar et al., 2011). The resultant high-quality reads were aligned using the fast, secondary-structure aware INFERNAL aligner (Nawrocki and Eddy, 2007). Their operational taxonomic units (OTUs) and rarefaction curves (Colwell and Coddington, 1994) were calculated at a 97% similarity level using the RDPipeline complete-linkage clustering tool. Shannon–Weaver (Shannon and Weaver, 1963), Chao1 richness (Chao and Bunge, 2002), and evenness indices were also calculated with the RDPipeline. Taxonomic classification of the reads was performed at the phylum and genus levels using the RDP Naïve Bayesian rRNA Classifier 2.5 trained on 16S rRNA training set 9 (Wang et al., 2007) with an 80% confidence threshold.

### Metabolite Analysis using ^1^H NMR Spectroscopy

We used ^1^H NMR spectroscopy to analyze *doenjang* metabolites across the entire fermentation period. Metabolites included monosaccharides, organic acids, and nitrogen compounds such as amino acids and BAs. To minimize quantification errors due to large particles, 10 g of *doenjang* samples were dried in an oven at 80°C for 1 h and ground into a fine power using a pestle and mortar. For sufficient metabolite extraction, 0.2 g of *doenjang* powder was resuspended in 1.5 mL of 99.9% deuterium oxide (D_2_O; Sigma–Aldrich, USA) containing 5 mM sodium 2,2-dimethyl-2-silapentane-5-sulfonate (DSS, 97%; Sigma–Aldrich) and incubated on ice with occasional shaking for 1 h. The *doenjang* powder solutions were centrifuged at 12,000 rpm and 4°C for 10 min, the 600 μL of the supernatant were transferred into NMR tubes. We obtained ^1^H NMR spectra with a Varian Inova 600-MHz NMR spectrometer (Varian, USA); *doenjang* metabolites were identified and quantified using the Chenomx NMR Suite program (version 6.1; Chenomx, Canada). Metabolite concentrations were calculated as μmol per g-dry weight *doenjang*.

### Sequencing Data Accession Number

The sequence data of the 16S rRNA genes from this study are publicly available in the NCBI Short Read Archive under accession no. SRP072427 (NCBI BioProject PRJNA315598).

## Results

### General Features of *Doenjang* Fermentation

**FIGURE 1:**
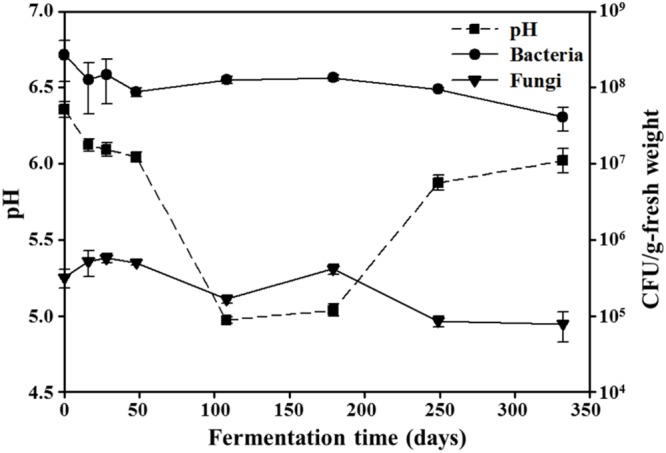
**Changes in pH, as well as bacterial and fungal abundances, during *doenjang* fermentation.** Data for pH and colony forming units (CFU) are presented as the means ± SD of *doenjang* samples in triplicate.

The initial pH values of the *doenjang* samples were approximately 6.4 (**Figure 1**). During the early fermentation period (0–48 days), pH decreased relatively slowly to approximately 6.0; after 48 days, their drop in pH occurred more quickly. From 108 to 179 days of fermentation, pH remained at around 5.0, after which the samples gradually became more basic again, reaching approximately 6.0 during the late fermentation period (249–332 days of fermentation).

Bacterial and fungal viable cells in *doenjang* were counted on their representative growth agar media, TSA, and PDA, respectively, (**Figure 1**); bacteria and fungi grown on PDA and TSA, respectively, were excluded from the counting by their colony morphologies. The initial bacterial cells were approximately 2.7 × 10^8^ CFU/g-fresh weight; as fermentation continued, bacterial cell numbers gradually decreased to approximately 4.1 × 10^7^ CFU/g-fresh weight. Similarly, fungal cell counts also experienced a gradual decrease, from an initial number of 3.2 × 10^5^ CFU/g-fresh weight to 7.9 × 10^4^ CFU/g-fresh weight over the course of fermentation. The NaCl concentrations remained relatively constant at approximately 17.5 ± 0.5% (w/w) during the entire fermentation period.

### Changes in Bacterial Diversity during *Doenjang* Fermentation

**FIGURE 2:**
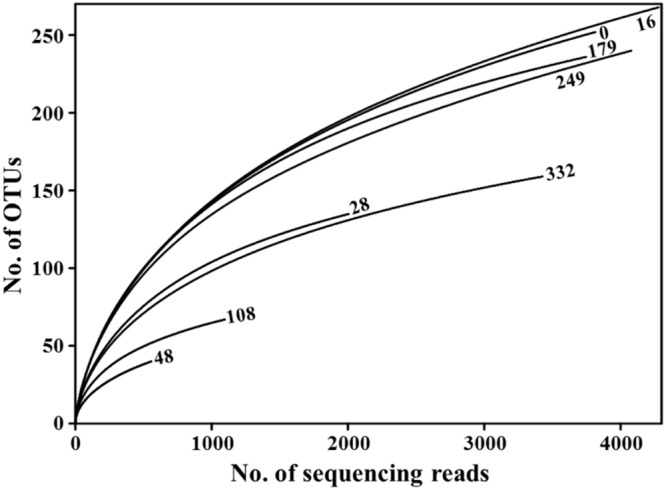
**Rarefaction curve analysis of 16S rRNA gene sequencing reads showing variation in bacterial diversity throughout the entire *doenjang* fermentation period.** Rarefaction curves were generated with the RDPipeline, using a 97% OTU (operational taxonomic units) cutoff value. Numbers next to the curves indicate *doenjang* sampling time (days).

We generated 43,432 sequencing reads from eight *doenjang* samples using barcoded pyrosequencing. After cleaning, 23,031 high-quality reads, with an average 472-bp length and 2,878 reads per sample, were obtained for the analysis of bacterial diversity and community (**Table 1**). The rarefaction analysis showed that bacterial diversity fluctuated slightly over the entire *doenjang* fermentation period (**Figure 2**), potentially indicating the active occurrence of bacterial succession. Bacterial diversity decreased during the early fermentation period (28 and 48 days) and increase after 48 days until 179 days, only to decrease again during the late fermentation period (249–332 days). All calculated diversity indices (OTU, Shannon–Weaver, Chao1, and evenness) supported the results of the rarefaction curve analysis, although the number of reads obtained affected the bacterial diversity indices (**Table 1**).

**Table 1:** Bacterial pyrosequencing data sets derived from the *doenjang* samples and their statistical diversity analysis.

Sample (day)	Total reads	High quality reads	OTUs^a^	Shannone–Weaver^a^	Chao1^a^	Evenness^a^
0	5296	3817	252	3.4	351.7	0.62
16	5877	4285	268	3.6	393.6	0.64
28	2783	2010	135	3.2	186.8	0.64
48	717	559	40	2.2	55.1	0.60
108	7520	1100	67	2.7	85.1	0.64
179	10816	3749	236	3.6	322.7	0.66
249	5704	4081	240	3.6	384.0	0.66
332	4719	3430	159	3.2	216.2	0.62

*OTUs, operational taxonomic units. ^a^Diversity indices were calculated using the RDPipeline tools.*

### Changes in Bacterial Community Composition during *Doenjang* Fermentation

**FIGURE 3:**
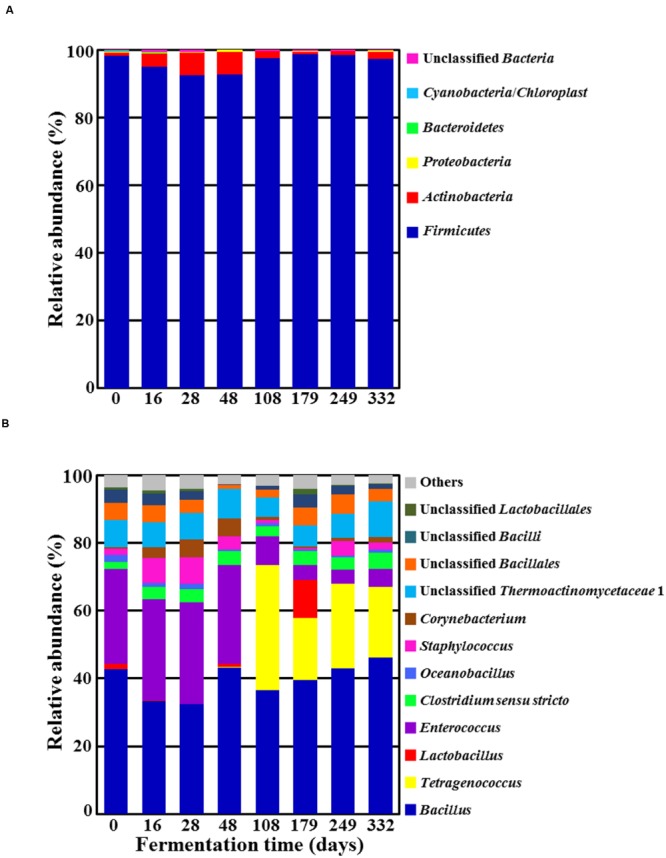
**Taxonomic classification at the phylum **(A)** and genus **(B)** levels showing bacterial community fluctuations during *doenjang* fermentation.** “Others” in **(B)** refers to genera exhibiting a read percentage<1.0% of the total reads in all *doenjang* samples.

Results from phylum- and genus-level classifications of high quality pyrosequencing reads are shown in **Figure 3**, demonstrating bacterial community fluctuations during *doenjang* fermentation. The phylum-level analysis revealed that only *Firmicutes* predominated during the entire fermentation period (**Figure 3A**). *Actinobacteria* was also detected as a minor group, with maximum relative abundance reaching approximately 6.5% at 48 days. The genus-level analysis revealed *Bacillus* to be predominant at a relative abundance of approximately 40%, without an evident fluctuation (**Figure 3B**). *Enterococcus* was also identified as a dominant group during early fermentation, but its relative abundance rapidly decreased with the sudden increase of *Tetragenococcus* at 108 day-*doenjang* samples. The latter genus was not observed initially but appeared as a dominant group from 108-day samples, and its high relative abundance lasted until the end of fermentation (day 332). Interestingly, *Lactobacillus* was identified as a dominant group in 179-day samples. Other minor bacterial genera detected in the *doenjang* samples included *Staphylococcus, Clostridium sensu stricto*, unclassified *Thermoactinomycetaceae* 1, and unclassified *Bacillales* from *Firmicutes*, as well as *Corynebacterium* from *Actinobacteria*; these groups did not exhibit dramatic fluctuations in their relative abundance during fermentation.

### Metabolite Changes during *Doenjang* Fermentation

**FIGURE 4:**
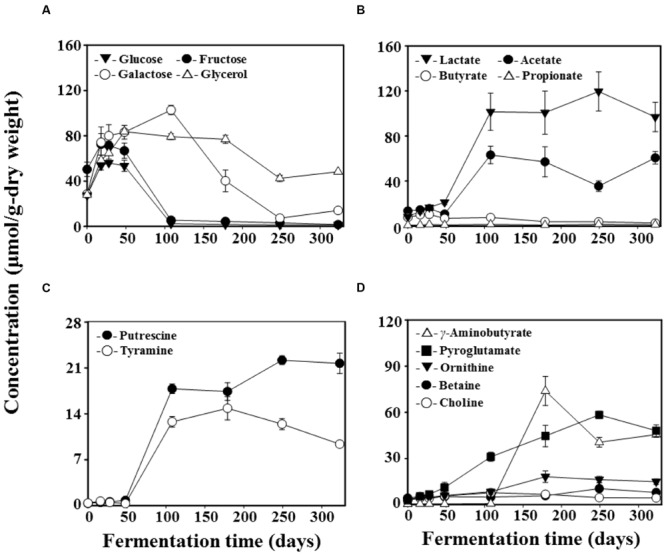
**Variation in the content of free organic compounds **(A)**, organic acids **(B)**, biogenic amines **(C)**, and nitrogen compounds **(D)** during *doenjang* fermentation.** Quantifications were performed in the Chenomx NMR suite program (version 6.1, Chenomx, Canada) with sodium 2,2-dimethyl-2-silapentane-5-sulfonate (DSS, 97%) as the internal standard. Data are presented as means ± SD.

Results from the ^1^H NMR analysis of metabolite content throughout *doenjang* fermentation are presented in **Figure 4**. Glucose, fructose, galactose, and glycerol were identified as the primary free organic compounds, and their levels increased quickly during the early fermentation period (**Figure 4A**). However, after approximately 16 days, glucose and fructose concentrations dropped by 108 days; they were almost entirely consumed. In contrast, galactose concentrations continued to increase until 108 days of fermentation, but then began to decrease from 179 days, finally approaching zero at 249 days. Glycerol concentrations reached maximum at 48 days and then experienced a gradual decrease until the end of fermentation.

Lactate and acetate were identified as the major organic acids during *doenjang* fermentation (**Figure 4B**). Both increased rapidly in 108-day samples and then exhibited fairly constant concentrations until the end of fermentation. Minor organic acids found to occur during *doenjang* fermentation were butyrate and propionate. Next, putrescine and tyramine were identified as the dominant BAs in *doenjang*; similar to lactate and acetate, these amines also increased rapidly in 108-day samples (**Figure 4C**). Nitrogen compounds detected in *doenjang* were ornithine, betaine, choline, γ-aminobutyric acid (GABA), and pyroglutamate (**Figure 4D**). Pyroglutamate increased continually until 249 days of fermentation. In particular, GABA exhibited a very rapid increase at 179 days.

**FIGURE 5:**
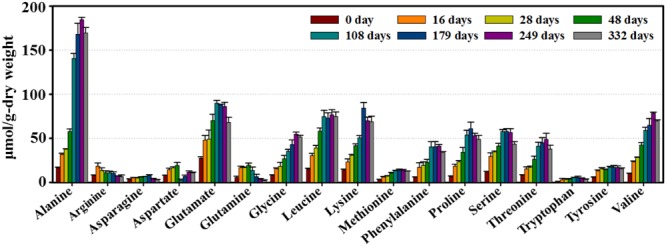
**Variation in major amino acids identified from *doenjang* samples during fermentation.** Quantifications were performed in the Chenomx NMR Suite (version 6.1, Chenomx, Canada) with sodium 2,2-dimethyl-2-silapentane-5-sulfonate (DSS, 97%) as the internal standard. Data are presented as means ± SD.

Finally, the amino acids well known as the main contributors to flavor and taste in *doenjang* products (Park et al., 2000, 2002; Kim and Lee, 2003) were also major metabolites in our samples (**Figure 5**). Amino acid concentrations rapidly increased during the early fermentation period. Most (alanine, glutamate, leucine, lysine, and phenylalanine) continued to increase until 108–249 days, and then gradually decreased throughout the remainder of the fermentation.

## Discussion

Traditional *doenjang* is produced by a long-term fermentation of solid parts obtained from a *doenjang*-*meju* and brine mixture, which is prepared by soaking the fermented *doenjang*-*meju* bricks in an 18–20% (w/v) solar salt solution for 40–60 days without the use of starter cultures. Because both bacteria and fungi are involved in *doenjang*-*meju* fermentation (Kim et al., 2010b; Lee J.H. et al., 2010; Jung et al., 2014) and have been detected in *doenjang* samples (Kim et al., 2009; Shukla et al., 2014), researchers have suggested that the two microbial groups may also play important roles in *doenjang* fermentation. We noted the presence of both bacteria and fungi in our *doenjang* samples (**Figure 1**). However, viable bacterial cell counts were approximately three orders of magnitude higher than fungal counts during fermentation. In addition, *doenjang* fermentation mostly occurs under anaerobic and high moisture conditions. Thus, fungi that prefer dry and aerobic conditions may exhibit lower metabolic activity compared with bacteria during this process. We therefore inferred that the observed fungal growth derived mostly from spores without metabolic activity that had originated in the *doenjang*-*meju*. This inference was supported by the lack of hyphae or mycelia in the *doenjang* samples (data not shown). These results suggest that bacteria are probably more responsible for *doenjang* fermentation than fungi. Hence, we chose to focus on bacteria in the microbial community analysis.

Although 16S rRNA gene sequencing is the most powerful tool in bacterial taxonomy, it is limitedly used at the species level classification due to the low phylogenetic resolution and poor discriminatory power of 16S rRNA gene sequence at the species level (Fox et al., 1992; Janda and Abbott, 2007). It has been generally accepted that it would be impossible to assign 16S rRNA gene sequences to the level of species because the taxonomies end at the level of genus and DNA relatedness studies are necessary to provide absolute resolution to these taxonomic problems (Schloss and Westcott, 2011). Therefore, in this study we taxonomically classified the 16S rRNA gene sequencing reads at the phylum and genus levels. The genera observed on day 0 of fermentation [*Bacillus, Enterococcus, Lactobacillus, Clostridium, Staphylococcus, Corynebacterium*, and *Oceanobacillus* (**Figure 3B**)] accorded well with genera detected in previous snapshot analyses of *doenjang* fermentation (Cho and Seo, 2007; Kim et al., 2009; Nam et al., 2012; Jeong et al., 2014). With the exception of *Oceanobacillus*, all genera had also been identified from *doenjang*-*meju* (Jung et al., 2014), despite the preparation step of soaking the bricks in solar salt solution for 42 days. These results suggest that *doenjang*-*meju* is a major source of bacteria in *doenjang* fermentation. In contrast, *Oceanobacillus* and *Tetragenococcus* (a dominant group at 108 days of fermentation) likely derived from solar salts because they were not found in *doenjang*-*meju* (Jung et al., 2014). Moreover, the two genera are halotolerant or halophilic groups that have been frequently isolated from high-saline environments, including solar salterns (Baati et al., 2010; Lee S.Y. et al., 2010). Thus, solar salts appear to be another important bacterial source in *doenjang* fermentation.

*Bacillus* was one of the predominant populations in *doenjang* during the entire fermentation period (**Figure 3B**). Due to the well-documented predominance of *Bacillus* in both culture-dependent and culture-independent studies on *doenjang*, researchers have generally assumed that this group is primarily responsible for the fermentation process (Yoo et al., 1999; Kim et al., 2010a; Jeon et al., 2016; Tamang et al., 2016b). However, members of *Bacillus* grow aerobically, and therefore, we consider it unlikely that they can be principal actors in *doenjang* fermentation, which occurs almost entirely under anaerobic conditions. Furthermore, most *Bacillus* members do not grow well under conditions more saline than 15% NaCl, although they have been detected from *doenjang* samples with ∼18% salt concentrations (Jung et al., 2014, 2016). Our data also showed that *Bacillus* abundance was relatively constant throughout fermentation and was unassociated with fluctuations in metabolite content (**Figure 4**). Therefore, we infer that *Bacillus* in *doenjang* are probably metabolically inactive or exist as spores. Although their relative abundance is high, we infer that this genus is not primarily responsible for *doenjang* fermentation.

*Enterococcus* are facultative anaerobic, Gram-positive, cocci-shaped lactic acid bacteria that occurred dominantly alongside *Bacillus* during the early fermentation period (**Figure 3B**). This finding corresponds to previous studies that have also reported *Enterococcus* as a dominant population in *doenjang* (Cho and Seo, 2007; Kim et al., 2009; Nam et al., 2012). Some members of *Enterococcus* are pathogenic and have caused infections in humans (Fisher and Phillips, 2009), but other species such as *E. faecalis* or *E. faecium* have been frequently isolated from fermented foods and even used as probiotics (M’hir et al., 2012; Todorov and Holzapfel, 2015). Therefore, the dominance of *Enterococcus* was expected and likely consisted of non-pathogenic species. The high counts of *Enterococcus* during the early fermentation period was associated with the increase of glucose, fructose, galactose, and glycerol (**Figures 3B and 4A**), which were probably generated through the hydrolysis of polysaccharides, flavonoid glycosides, and lipids in *doenjang*-*meju*. However, most *Enterococcus* members do not show glyosidic and lipase activities under high saline conditions such as *doenjang* (Jeong et al., 2014), suggesting that their metabolism was too low during fermentation to hydrolyze the organic carbon compounds. Instead, we speculated that endogenous enzymes derived from *doenjang*-*meju* might be responsible for the observed increases in glucose, fructose, galactose, and glycerol. Moreover, despite the dominance of *Enterococcus*, we only observed slight increases in lactate and acetate during early fermentation, a finding that supports the hypothesis of weak metabolic activity in these lactic acid bacteria (**Figures 3B and 4B**). However, additional studies will be necessary to investigate how organic carbon compounds increase in *doenjang* during the early fermentation period.

Bacterial community analysis showed that *Staphylococcus* increased to approximately 7.6% of the total bacterial abundance at 28 days of fermentation (**Figure 3B**). Similar to *Enterococcus*, some members of this genus are pathogenic, but other *Staphylococcus* species are considered starters for fermentation and accordingly, have been identified from several fermented foods, including *doenjang*, sausage, and fish sauce (Fontana et al., 2005; Yongsawatdigul et al., 2007; Kim et al., 2009; Guan et al., 2011; Jung et al., 2013; Lee et al., 2015). Therefore, the *Staphylococcus* species observed in our *doenjang* samples probably do not have pathogenic properties, but additional research is required to understand their exact role during fermentation, as the function of *Staphylococcus* was unclear in this study.

Bacterial community analysis demonstrated that *Tetragenococcus*, a genus of halophilic, Gram-positive lactic acid bacteria, was a dominant group in 108-day samples (**Figure 3B**). The dominance of *Tetragenococcus* corresponded well to the rapid increases in lactate and acetate concentrations, as well as the decreases in glucose and fructose, occurring around the same time (**Figures 3B and 4A,B**). Additionally, the drop in pH at 108 days was in line with heightened lactate and acetate production (**Figure 1**). Together, these results suggest that *Tetragenococcus* may be primarily responsible for the production of organic acids during *doenjang* fermentation. BAs, including putrescine, cadaverine, spermidine, histamine, and tyramine, are low-molecular-weight nitrogenous organic compounds produced via microbial decarboxylation of amino acids and nitrogen compounds during food fermentation (Shalaby, 1996; Park et al., 2000; Moon et al., 2010; Costantini et al., 2013; Jung et al., 2015). These compounds are known to be produced in *doenjang* (Shukla et al., 2010; Kim and Ji, 2015), but the agents of their production are unknown. The increase of putrescine and tyramine, respectively, generated through ornithine and tyrosine decarboxylation, was also associated with *Tetragenococcus* dominance (**Figures 3B and 4C**). These results suggest that *Tetragenococcus* may be a key agent in biogenic-amine production during *doenjang* fermentation. Previous studies have also shown that members of *Tetragenococcus* play important roles in the fermentation of salted goods, including fermented seafood; thus, this genus may be a good bacterial starter for flavor enhancement of fermented foods (Chen et al., 2006; Udomsil et al., 2011; Kim and Park, 2014; Jung et al., 2015, 2016). However, some *Tetragenococcus* species appear to produce BAs primarily via plasmid-encoded decarboxylation genes (Satomi et al., 2008, 2011, 2014), which increases the difficulty of using *Tetragenococcus* as starters for salted food fermentation. To address this problem, strains without the ability to produce these compounds have been applied to control biogenic-amine generation during fermentation (Udomsil et al., 2011; Kuda et al., 2012).

Interestingly, *Lactobacillus*, a group of Gram-positive, facultative anaerobic or microaerophilic, rod-shaped lactic acid bacteria, was identified as a dominant group in 179-day samples (**Figure 3B**). The non-protein amino acid GABA is a major inhibitory neurotransmitter and is produced through the irreversible α-decarboxylation of L-glutamate by glutamate decarboxylase. *Lactobacillus* species have been reported as major GABA-producing bacteria during fermentation (Makarova et al., 2006; Cho et al., 2007; Park and Oh, 2007; Siragusa et al., 2007; Komatsuzaki et al., 2008; Jeong et al., 2013). Our metabolite analysis supported these previous findings; galactose decreases and GABA increases were associated with the increase of *Lactobacillus* (**Figures 4A,D**), implying that members of *Lactobacillus* are responsible for GABA production during fermentation. Additionally, the ability of *Lactobacillus* to metabolize galactose is well-established and members of *Lactobacillus* have been detected in *doenjang* (Cho and Seo, 2007; Nam et al., 2012), indicating that lactate production from galactose and GABA synthesis by *Lactobacillus* are important processes during *doenjang* fermentation although no report showing their growth in 18% NaCl conditions exists.

To the best of our knowledge, this was the first study to investigate fluctuations in microbial communities and metabolite production simultaneously throughout the entire *doenjang* fermentation period. Ours was also the first to use *doenjang*-*meju* of known bacterial community composition in a study of *doenjang* fermentation. Here, we suggested that both *doenjang*-*meju* and solar salts are important bacterial sources in *doenjang* fermentation. Furthermore, we proposed that despite their overall abundance, *Bacillus* may be not as central to *doenjang* fermentation as previously assumed. Additionally, we showed that solar-salt-derived *Tetragenococcus* appears to be a primary producer of organic acids and BAs during *doenjang* fermentation, suggesting that *Tetragenococcus* strains without this ability are usable as starters, in order to reduce biogenic-amine concentrations. Finally, our results suggested that *Lactobacillus* is probably one of the major GABA producers during *doenjang* fermentation. In conclusion, this study contributed to an improved understanding of the biochemical processes underlying *doenjang* fermentation through exploring the functional properties of major *doenjang* microbial communities. The data generated should pave the way for additional research employing “omics” technologies, including metagenomics, metatranscriptomics, and metabolomics, which are certain to provide further insights into the production of safe and high-quality *doenjang*.

## Author Contributions

CJ conceived the ideas and supervised the work WJ and JJ developed the concepts and performed the experiments WJ and HL analyzed the data and CJ and WJ wrote the manuscript. The manuscript has been reviewed and edited by all authors.

## Conflict of Interest Statement

The authors declare that the research was conducted in the absence of any commercial or financial relationships that could be construed as a potential conflict of interest.

## Footnotes

1 http://pyro.cme.msu.edu/
