# Supplementary material for: Functional Characterization of Bacterial Communities Responsible for Fermentation of Doenjang: A Traditional Korean Fermented Soybean Paste
Source: Front Microbiol. 2016 May 31;7:827. doi: 10.3389/fmicb.2016.00827 (PMC4885865; doi:10.3389/fmicb.2016.00827)
Supplement: Supplementary file 1 [file Data_Sheet_1.DOCX]

**SUPPLEMENTARY INFORMATION**

**Functional characterization of bacterial communities responsible for fermentation of *doenjang*, a traditional Korean fermented soybean paste**

Woo Yong Jung†, Ji Young Jung†, Hyo Jung Lee, and Che Ok Jeon*

*Department of Life Science, Chung-Ang University, Seoul 06974, Republic of Korea*

†These authors contributed equally to this study.

*Corresponding author: Che Ok Jeon

| **Supplementary Table S1.** Adaptor and barcode sequences in PCR primer sets | |
| --- | --- |
| Name | Sequence (5'-3') |
| **Adapters** |  |
| A adapter | CCATCTCATCCCTGCGTGTCTCCGACTCAG |
| B adapter | CCTATCCCCTGTGTGCCTTGGCAGTCTCAG |
| **Fermentation time (days)** |  |
| 0 | AGAGCTG |
| 16 | ACTGAGT |
| 28 | CGATGAG |
| 48 | CTGTGAT |
| 108 | CACACGATAG |
| 179 | ATGTACGATG |
| 249 | ATCGTAGCAG |
| 332 | ATCACGTGCG |
